# Targeting the Mincle and TLR3 receptor using the dual agonist cationic adjuvant formulation 9 (CAF09) induces humoral and polyfunctional memory T cell responses in calves

**DOI:** 10.1371/journal.pone.0201253

**Published:** 2018-07-31

**Authors:** Aneesh Thakur, Athina Andrea, Heidi Mikkelsen, Joshua S. Woodworth, Peter Andersen, Gregers Jungersen, Claus Aagaard

**Affiliations:** 1 Section for Immunology and Vaccinology, National Veterinary Institute, Technical University of Denmark, Kgs. Lyngby, Denmark; 2 Department of Infectious Disease Immunology, Statens Serum Institut, Copenhagen, Denmark; Instituto Butantan, BRAZIL

## Abstract

There is a need for the rational design of safe and effective vaccines to protect against chronic bacterial pathogens such as *Mycobacterium tuberculosis* and *Mycobacterium avium* subsp. *paratuberculosis* in a number of species. One of the main challenges for vaccine development is the lack of safe adjuvants that induce protective immune responses. Cationic Adjuvant Formulation 01 (CAF01)—an adjuvant based on trehalose dibehenate (TDB) and targeting the Mincle receptor—has entered human trials based on promising pre-clinical results in a number of species. However, in cattle CAF01 only induces weak systemic immune responses. In this study, we tested the ability of three pattern recognition receptors, either alone or in combination, to activate bovine monocytes and macrophages. We found that addition of the TLR3 agonist, polyinosinic:polycytidylic acid (Poly(I:C)) to either one of the Mincle receptor agonists, TDB or monomycoloyl glycerol (MMG), enhanced monocyte activation, and calves vaccinated with CAF09 containing MMG and Poly(I:C) had increased cell-mediated and humoral immune response compared to CAF01 vaccinated animals. In contrast to the highly reactogenic Montanide ISA 61 VG, CAF09-primed T cells maintained a higher frequency of polyfunctional CD4+ T cells (IFN-γ^+^ TNF-α^+^ IL-2^+^). In conclusion, CAF09 supports the development of antibodies along with a high-quality cell-mediated immune response and is a promising alternative to oil-in-water adjuvant in cattle and other ruminants.

## Introduction

Vaccines are the most efficient tool for preventing diseases caused by infectious pathogens. Many of the current vaccines were developed fifty or more years ago and are based on live attenuated forms of the pathogen. For intracellular mycobacterial infections, there is a strong need for modern vaccines not only for humans but also for a number of other species including, cattle, goat, sheep, buffalo, and deer. The current challenge is to achieve a potent vaccination effect specific for the intracellular mycobacterial infection while avoiding reactogenicity and toxicity typically associated with the most potent adjuvants, and without interfering with the diagnostic tests currently in place for these infections [1]. Subunit vaccines based on adjuvant formulations such as cationic adjuvant formulation 01 (CAF01) combined with selected antigens seems well suited for this. CAF01 is based on the cationic lipid DDA (dimethyldioctadecylammonium) and TDB (α, α´ trehalose dibehenate). DDA´s function is to create a long-lasting depot at the site of injection and increase cellular uptake of antigens. TDB stabilizes DDA liposomes and is an agonist of the macrophage inducible C-type lectin (Mincle) receptor that activates antigen-presenting cells through the TLR-independent Syk-CARD9 pathway [2]. Mouse models have shown that CAF01 induces a Th1- and Th17-biased CD4 T cell response combined with a humoral immune response [3] and confers protective immunity against tuberculosis (TB) in mice, guinea pig and non-human primate models when formulated with antigens from *Mycobacterium tuberculosis* [4–6]. Furthermore, CAF01-adjuvanted vaccines have shown promising results against chlamydia, malaria, and influenza infections in animal models [4, 7–9]. CAF01 has been tested successfully in Phase I clinical trials where the safety, tolerability, and immunogenicity profile of the adjuvant was investigated when administered in combination with both a protein TB vaccine (*ClinicalTrials*.*gov identifier NCT00922363*) and a peptide based HIV-1 cocktail (*ClinicalTrials*.*gov identifier NCT01141205*). Thus, the use of CAF01 seems to be quite versatile. Unfortunately, immunization studies in cattle have shown that CAF01 only induces a weak CD4 T cell response against the same TB subunit vaccine (Ag85B-ESAT6) that gave strong responses in other species [10]. Using other mycobacterial antigens, we have confirmed the low immunogenicity of CAF01 in cattle. However, we also found that both the age of the animal at immunization and the antigen itself had a major influence on the magnitude of the specific response [11]. In larger animals, there is very limited studies regarding vaccine dose and ratio of antigen and adjuvant and it is possible that increasing the dose or changing the ratio could improve immunogenicity but may be at an inhibitory cost. With the aim of improving vaccine efficacy in cattle, we test the ability of selected immune modulators to activate monocytes isolated from blood samples from naïve animals. We find that by combining TDB or MMG with a double stranded RNA analog polyinosinic:polycytidylic acid (Poly(I:C)) that activates APCs via toll like receptor 3 (TLR3) we obtain a synergistic effect on the in vitro activation of bovine monocytes. This translates into increased humoral and cell-mediated responses in calves immunized with the newly developed CAF09 adjuvant containing DDA, MMG, and poly(I:C), and we compare the responses to a water-in-oil based adjuvant developed for veterinary use (Montanide^™^ ISA 61 VG) [12, 13].

## Materials and methods

### Animals

Male jersey calves (n = 12) were obtained from a dairy farm proven to have a true prevalence equal or close to zero by the Danish paratuberculosis surveillance program [14] and were included at two months of age. All animals used in the study were kept under appropriate bio containment facilities (biosafety level 2 [BSL-2]) in a community pen with straw bedding. Animals were fed pellets and hay ad libitum and bedding was changed every day. The use of animals in this study was approved by the Danish National Experiment Inspectorate and work was carried out in accordance with their regulations and policies, following institutional ethical guidelines.

### Isolation of bovine CD14 positive peripheral blood mononuclear cells

Peripheral blood mononuclear cells (PBMCs) were isolated by density gradient centrifugation using blood collected aseptically in heparinized tubes. Briefly, red blood cells were lysed by adding 10 ml 0.84% w/v NH_4_Cl to 20 ml blood in 50 ml tubes. After 2 min. on ice, 20 ml ice cold PBS was added and the cells were pelleted (10 min, 1800 rpm, 4°C). Cell pellet was re-suspended in 35 ml PBS and layered over 15 ml Lympholyte^®^ Mammal (Cedarlane Laboratories, Burlington, NC, USA) at room temperature. After centrifugation (10 min, 2500 rpm, 15°C) the PBMC interface between Lympholyte^®^ and PBS was harvested and washed twice before being re-suspended in 1 ml PBS. CD14 (cluster of differentiation 14) positive cells were purified by positive selection using the human CD14 MACS^®^ cell separation kit (Miltenyi Biotec GmbH), according to the manufacturer’s protocol. In brief, a single cell suspension of PBMCs was generated by filtering through a 30 μm pre-separation filter whereafter cells were incubated with magnetic beads conjugated to mouse anti-human CD14 monoclonal antibodies, which cross react with the bovine CD14 molecules. The CD14+ monocytes were retained on a magnetic column.

### TDB and MMG lipid layers

Solutions of α,α‘-trehalose 6,6’- dibehenate and (TDB) and Monomycolyl glycerols (MMG) lipids were prepared in methanol/chloroform and chloroform respectively to a concentration of 1 mg/ml, in glass vials (1 mg/vial). To evaporate the solvent, the solutions were exposed to a stream of nitrogen gas for one hour followed by overnight incubation in the fume hood. The dry lipids were stored at -20°C or used directly. Serial dilutions of the dry lipid stock were performed in 96 well, flat bottom plates using isopropanol as solvent. After evaporation of the isopropanol, the lipid-coated plates were used for cell stimulation.

### In vitro stimulation of monocytes with immunostimulators

Purified CD14 positive monocytes (5*10^4^ cells/well) were added to 96 well flat bottom plates, that were either pre-coated with lipid layers of TDB or MMG or supplemented with 5 or 50 μg/ml Poly(I:C) or 15, 5 or 1.7 μg/ml CAF09 [3]. All combinations of immunostimulators were tested in triplicates. After 3 days stimulation, the interleukin-6 levels (IL-6) were determined in cell supernatants by enzyme-linked immunosorbent assay (ELISA) (Thermo Fisher Scientific, US) according to the manufacturer’s protocol (data in S1 Table).

### Antigens, adjuvants and immunization

All animals were immunized with antigenic *Mycobacterium avium* subsp. *paratuberculosis* (MAP) proteins in a mixture comprising of MAP3694c (20 μg/vaccination) and a fusion protein (30 μg/vaccination) consisting of the proteins: MAP1507, MAP1508, MAP3783 and MAP3784. The vaccine antigens were produced as recombinant proteins in *E*. *coli* and purified by metal affinity and anion columns as previously reported [14]. One hour prior to vaccination, the antigens were formulated with adjuvant. For Montanide^™^ ISA 61 VG (Seppic, France), a mineral water-in-oil based adjuvant, antigens, sterile Tris buffer pH 7.8 and adjuvant were mixed in the recommended ratio and the formulation passed 20 times slowly and then 60 times at high speed through a syringe-connector-syringe apparatus supplied with the adjuvant. The cationic-liposome adjuvants CAF01 (DDA, 2500 μg/ml and TDB, 500 μg/ml) and CAF09 (2500 μg/ml DDA, 500 μg/ml TDB and 500 μg/ml Poly(I:C) were prepared as previously described [4, 11, 15] and mixed with antigens in Tris buffer at room temperature one hour before use. All vaccines were administered as 2 ml subcutaneous injections in the mid-neck region at experimental week 0 and 4. Control groups were immunized twice with Tris buffer containing antigens alone. Two independent immunization studies were performed (Table 1).

**Table 1:** Experimental design.

Experiment No. and adjuvant group	Number of animals	Immunization	Immunization schedule (wks)
**Exp. 1**			
ISA 61 VG	2	ISA 61 VG + MAP proteins	0 and 4
CAF01	2	CAF01 + MAP proteins	0 and 4
No adjuvant	2	Tris buffer + MAP proteins	0 and 4
**Exp. 2**			
ISA 61 VG	1	ISA 61 VG + MAP proteins	0 and 4
CAF01	1	CAF01 + MAP proteins	0 and 4
CAF09	3	CAF09 + MAP proteins	0 and 4
No adjuvant	1	Tris buffer + MAP proteins	0 and 4

### Isolation of peripheral blood mononuclear cells

PBMCs for restimulation assays described below were isolated from heparinized cattle blood by Ficoll^®^ Paque Plus 1.077 (GE Healthcare Life Sciences, US) gradient density centrifugation. For ex vivo IFN-γ ELISPOT assay, cells were washed and resuspended in serum-free medium (AIM-V^®^, Invitrogen). For intracellular cell staining, cells were washed twice in RPMI 1640 media (Life Technologies, UK) supplemented with 2% heat-inactivated fetal calf serum (Sigma Aldrich). After washing, cells were resuspended in RPMI 1640 medium supplemented with 10% heat-inactivated fetal calf serum, 100 U/ml penicillin (Gibco, UK), and 100 μg/ml streptomycin sulphate (Gibco, UK).

### Whole-blood IFN-γ assay

Cytokine IFN-γ production in antigen-stimulated whole-blood cultures was determined by culturing whole-blood in the presence of antigens as described previously [11]. Briefly, 0.5 ml whole-blood was cultured in the presence of 1 μg/ml of fusion protein, MAP3694c, MAP2487c (non-vaccine control protein), 10 μg/ml of Johnine purified protein derivative (PPDj), PBS, or positive control stimulation with 1 μg/ml superantigen *Staphylococcus* enterotoxin B (SEB). Secreted IFN-γ was measured in cell supernatants using an in-house monoclonal sandwich ELISA as previously described [11]. To define the antigen-specific IFN-γ response the background level (IFN-γ response to PBS) was subtracted from the response to the vaccine antigens (data in S2 Table).

### Ex vivo IFN-γ ELISPOT assay

An in-house developed and standardized ex vivo IFN-γ ELISPOT assay was used to enumerate effector T cells as a function of IFN-γ production. Briefly, ELISPOT plates (EMD Millipore) were coated overnight at 4°C with bovine IFN-γ-specific monoclonal antibody (clone 6.19) at a concentration of 5 μg /ml. After washing with sterile PBS, wells were blocked with serum-free medium (AIM-V^®^, Life Technologies) and PBMCs suspended in serum-free medium were added (1 x 10^6^ PBMC/well) and cultured in the presence of fusion protein and MAP3694c antigen or mitogen SEB or a non-vaccine control protein MAP2487c at 1 μg/ml for 20 hours in a humified 37°C, 5% CO_2_ incubator. Plates were washed twice with distilled water and three times with PBS-0.01% Tween 20 (PBS-T). An anti-IFN-γ biotinylated monoclonal antibody (clone cc302) was added at a concentration of 1 μg /ml in PBS-0.1% bovine serum albumin (1 hour at room temperature). After washing four times with PBS-T, plates were incubated with streptavidin-alkaline phosphatase (Roche; 1:2000 dilution, 1 hour at room temperature) followed by washing three times with PBS-T and twice with sterile PBS. Spots were visualized using BCIP/NBT substrate (Sigma) and counted using an ELISPOT reader (AID GmbH)(data in S2 Table).

### Antigen-specific IgG1 ELISA

Serum levels of antigen-specific immunoglobulin G1 (IgG1) were measured by an in-house developed and standardized ELISA as previously described [11]. Briefly, plates were coated with 1 μg/ml of each of the five vaccine antigens or a non-vaccine MAP protein (MAP2487c, negative control). Serum samples were plated at 1:40 dilution. IgG1 was detected with HRP-conjugated mouse anti-bovine IgG1 mAb (clone IL-A60; Bio-Rad) diluted 1:500. Substrate was o-phenylenediamine dihydrochloride and optical density was read at 493 nm with a 649-nm reference subtraction (data in S3 Table). The results are presented as calibrated OD (ODc) as previously described [11].

### Cell stimulation, antibody staining and flow cytometry

Purified PBMCs (2 x 10^6^ cells/ml) were cultured in the presence of 1 μg/ml of fusion protein and 1 μg/ml MAP3694c protein for 6 hours followed by 16 hours in the presence of Brefeldin A (10 μg/ml; Sigma) and Monensin (2 μM/ml; Sigma). Control cultures containing either medium alone or mitogen (1 μg/ml of SEB) were run in parallel. After washing cultured PBMCs with PBS (supplemented with EDTA and sodium azide) the cells were stained. Anti-CD4 antibody (clone IL-A11; Veterinary Medical Research & Development, VMRD, Pullman, WA, USA) was used for surface stain in combination with violet dead cell stain (Invitrogen) before the cells were intracellularly stained with antibodies specific for IFN-γ (PE-conjugated, clone CC302; Bio-Rad), TNF-α (AF488-conjugated, clone CC327; Bio-Rad), and IL-2 (DyLight649-conjugated, Clone 86; a kind gift from Adam Whelan, APHA, UK) [16]. Using a BD FACSCanto II analyser equipped with 405, 488 and 633 nm lasers (BD Bioscience, USA) flow data were acquired and analyzed with BD FACSDiva software vs. 6.1.2 (data in S4 Table).

### ID Screen^®^ paratuberculosis indirect ELISA

For detection of anti-MAP antibodies, a commercial indirect ELISA kit (ID Vet, France) developed for the surveillance of MAP infection was used. Sera samples collected 7½ weeks after first vaccination were tested for total IgG production following manufacturer instructions. Results were reported as sample to positive ratio (S/P ratio) calculated using the formula S/P ratio = ((OD_Sample_−OD_Negative control_) ÷ (OD_Positive control_−OD_Negative control_) × 100). Animals were assigned MAP status as seropositive or seronegative according to manufacturer’s interpretations, with serum samples interpreted as negative if S/P ratio is ≤ 60, doubtful if between 60 and 70, and positive if ≥ 70 percent.

### Comparative tuberculin skin test

To rule out cross-reactivity with surveillance testing for bovine tuberculosis, comparative intradermal tuberculin testing was performed 8 weeks after first vaccination as previously described [11] and results were interpreted according to standard protocol (European Communities Commission regulation 141 number 1226/2002) [11].

### Statistical analysis

Data were analyzed using the Graphpad Prism 7 statistical package (GraphPad Software Inc., USA). Immune responses in all animal groups were compared by two-way analysis of variance (ANOVA) using time and vaccination as independent variables. For multiple comparison we used Tukey’s multiple comparison test, testing for both vaccine effects measured over the entire length of the experiment as well as effects at the individual time points. P values < 0.05 were considered significant.

## Results

### Synergistic effect on cell activation by combining receptor agonists is augmented by liposome formulation

**Fig 1:**
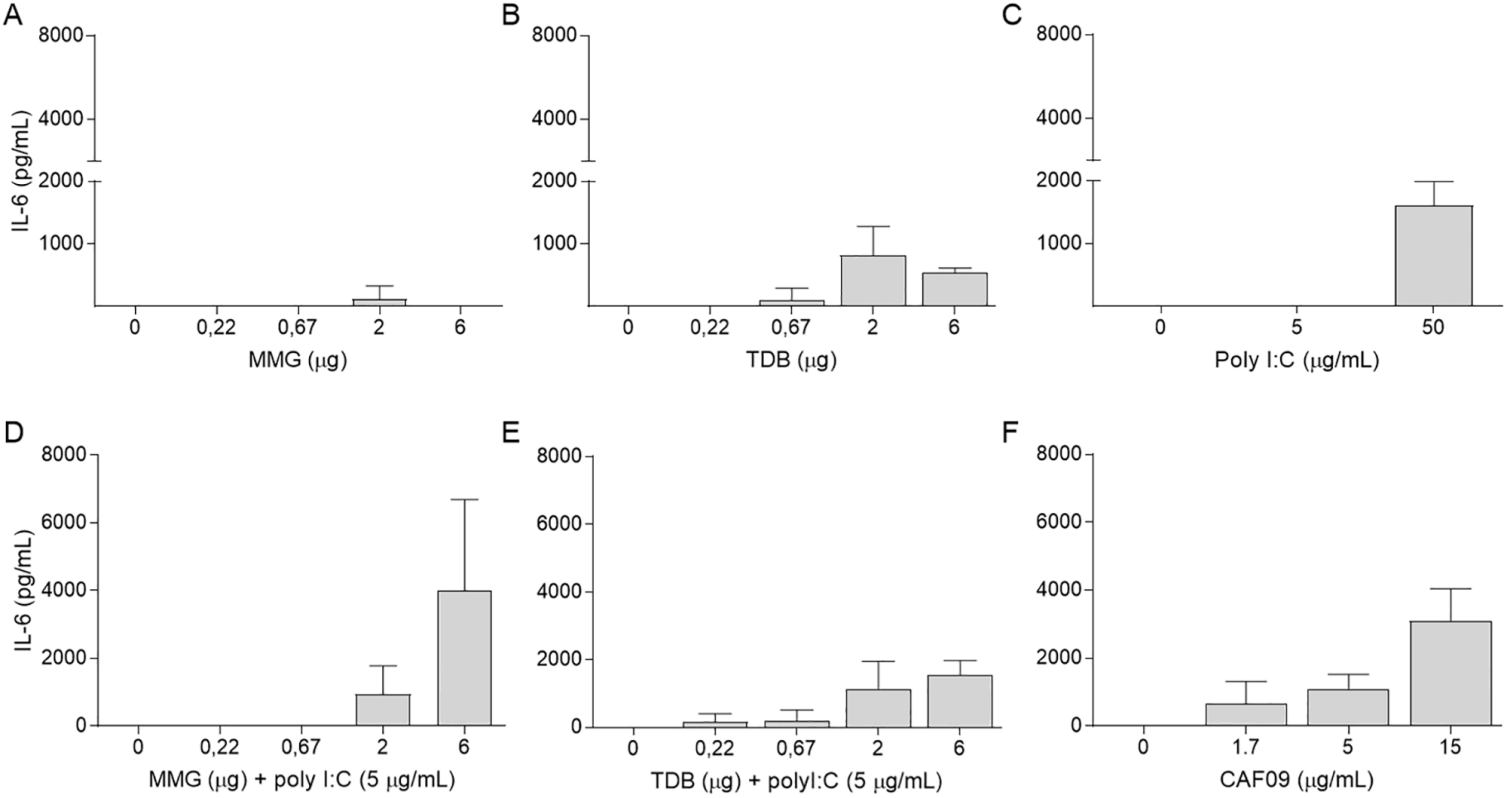
Agonists have synergistic effect on monocyte and macrophage activation in vitro. CD14+ cells were purified from peripheral blood mononuclear cells (PBMCs) and transferred to cell culture plates pre-coated with lipid layers of TDB (trehalose dibehenate) or MMG (monomycoloyl glycerol) or supplemented with Poly(I:C) (polyinosinic:polycytidylic acid) or CAF09. Levels of IL-6 (pg/ml) were measured in culture supernatants after three days of incubation by enzyme-linked immunosorbent assay (ELISA). Bars represent mean ± SD of three calves.

Using IL-6 secretion as a marker for monocyte and macrophage activation, we investigated the ability of three different immune modulators to activate CD14-purified bovine monocytes and macrophages *in vitro*. The immune modulators included two lipids, TDB and MMG, and a synthetic analog of double-stranded RNA, Poly(I:C). These lipids and the RNA analog are being exploited as immune stimulators in the CAF01, CAF04, and CAF09 adjuvants. CAF01 is based on the surfactant DDA and the glycolipid TDB. CAF04 is based on DDA and mycobacterial cell wall lipid MMG while CAF09 is based on DDA/MMG and poly(I:C). Both TDB and MMG lipids were capable of stimulating the CD14+ cells (Fig 1A and 1B). The stimulatory effect of the MMG lipid was quite small and only detectable at one MMG lipid coating density (2 μg/well). For TDB, the strongest response was measured at the same lipid coating density, but the level of released IL-6 cytokine was almost seven times higher than for MMG with monocyte activation for TDB lipid coating densities detected above and below the optimum. For soluble Poly(I:C), the lowest concentration tested (5 μg/ml) did not activate the CD14+ cells to produce and release IL-6, but increasing the Poly(I:C) concentration ten-fold (50 μg/ml) resulted in cell expression and secretion of relatively large quantities of IL-6 (1605 ± 384 pg/ml)(Fig 1C). To identify a possible synergistic effect from simultaneously stimulating the cells with two agonist binding to distinct receptors and activating different signaling pathways we combined the TLR3 activator, Poly(I:C), with either one of the two Mincle activators, TDB and MMG. When co-stimulated, the low concentration of Poly(I:C) (5 μg/ml) in the culture medium was sufficient to boost the cell activation and extend the activation range for both TDB and MMG lipids (Fig 1D and 1E). The co-stimulation of increasing TDB lipid with fixed Poly(I:C) concentration gave an increasing dose-response curve with IL-6 release reaching the same level as the high Poly(I:C) concentration for the highest TDB density (1542 ± 436 pg/ml). MMG and Poly(I:C) co-stimulation resulted in more than a doubling of IL-6 release into the medium, relative to TDB and Poly(I:C), for the highest lipid coating densities (3988 ±2701 pg/ml). Because MMG and Poly(I:C) co-stimulation induced a strong cell activation and CAF09 can induce both CD4+ and CD8+ cell mediated immunity combined with a humoral response in mice, we decided to test the ability of CAF09 adjuvant to activate bovine monocytes and macrophages in vitro (Fig 1F). CAF09 stimulation of bovine monocytes and macrophages resulted in a dose-response curve with the maximum activation (3090 ± 954 pg/ml IL-6) at the highest concentration tested (15 μg/ml CAF09). For comparison, this corresponds to an MMG dose of 0.43 μg and a Poly(I:C) concentration of 2.14 μg/ml, significantly below the MMG density (2 μg/well) or Poly(I:C) concentration (5 or 50 μg/ml) needed for cell activation when these were used individually or in combination without DDA liposomes. Based on the promising results we decided to test CAF09 as an adjuvant for use in cattle and compare it against CAF01 and the water-in-oil emulsion Montanide ISA 61 VG.

### Specific cell-mediated responses in calves after vaccination with the liposome based CAF09 adjuvant

**Fig 2:**
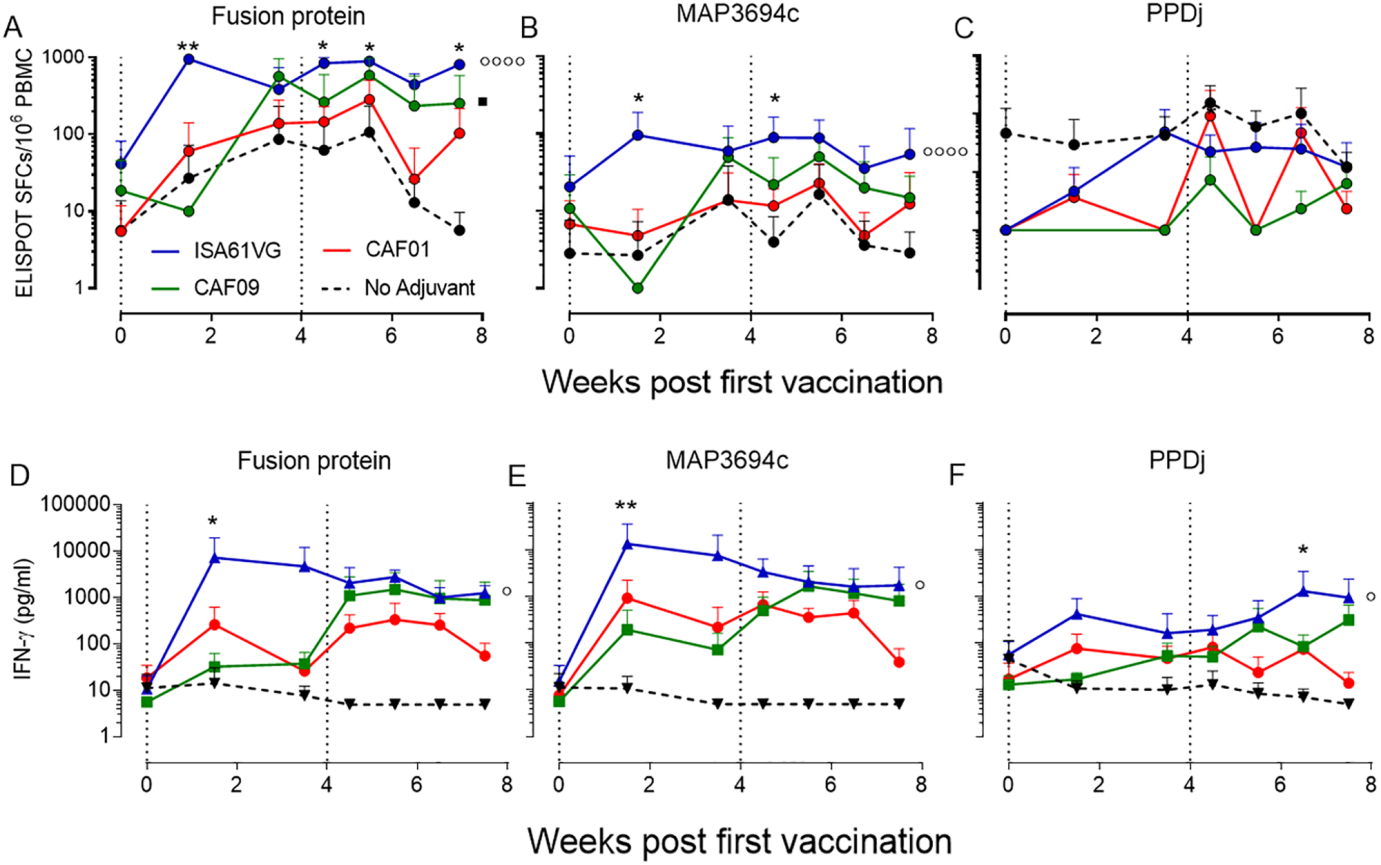
CAF09 and ISA 61 VG adjuvants induce a specific cell-mediated immune response. IFN-γ ELISPOT analysis (**A-C**) and IFN-γ ELISA (**D-F**) were performed on peripheral blood mononuclear cells (PBMCs) from vaccinated animals up to 7½ weeks after first immunization. Animals were immunized twice by the subcutaneous route with recombinant antigens and one of the indicated adjuvants. Vaccination time points are indicated by dotted lines. The frequency of vaccine-induced IFN-γ cells and released cytokine IFN-γ into the cell medium was determined after 24 hours of in vitro stimulation of PBMCs with either the vaccine fusion protein, MAP3694c or PPDj. Shown are means ± SD of three calves. Statistical analysis: two-way analysis of variance (ANOVA) and Tukey’s post-test. Black stars indicate significant difference relative to the non-adjuvant group. Circles and squares indicate differences between the curves for the adjuvanted and non-adjuvanted groups *p < 0.05, **p < 0.01, ****p < 0.001.

To monitor the development of cell-mediated responses with the different adjuvants, calves were immunized with mycobacterial protein antigens (a fusion protein consisting of MAP1507-MAP1508-MAP3783-MAP3784 and MAP3694c as a single protein) in the water-in-oil emulsion Montanide ISA 61 VG or the cationic liposome adjuvant formulations CAF01 and CAF09. At different time points, up to 7½ weeks after first immunization, blood was drawn and stimulated in vitro with vaccine antigens, whereafter the frequency of antigen-specific cells was measured by IFN-γ ELISPOT, and total IFN-γ secretion into the cell medium was measured by ELISA (Fig 2A–2F). Vaccination with antigens in Montanide ISA 61 VG resulted in a statistically significant and rapid induction of specific cells after one immunization (Fig 2A–2C). The number of IFN-γ producing cells were statistically higher than in CAF01 or non-adjuvant vaccinated animals regardless if we compared for whole fusion protein- or MAP3694c-specific cells (p<0.001). In CAF09 vaccinated animals the number of fusion protein-specific cells was higher than in the non-adjuvant group (p<0.05) but there was no statistical difference when compared to the CAF01 vaccination group. In the Montanide ISA 61 VG and CAF09 comparison, there was only a significant difference at the first time point after one vaccination where the CAF09-vaccinated animals had not yet developed a vaccine response. We found one animal in each of the CAF01 and Montanide ISA 61 VG adjuvant groups with a high PPDj response relative to the other animals. The secretion of IFN-γ in culture supernatants after antigen stimulation confirmed the patterns of vaccine-induced antigen-specific responses. Montanide ISA 61 VG induced statistically significant IFN-γ levels compared to the CAF adjuvants (p<0.01) and the non-adjuvant control group (p<0.05) before the second vaccination (Fig 2D and 2E). Interestingly, after a booster immunization the CAF09-induced IFN-γ level increased significantly and was nearly on par with the Montanide ISA 61 VG-promoted response throughout the rest of the study for both the fusion and MAP3694c protein. Accordingly, CAF09-induced IFN-γ responses measured at time points after the second immunization were statistically different from the responses in the non-adjuvanted control group (p<0.01) but, as with the ELISPOT data, were not statistically distinguishable from the other vaccination groups. IFN-γ release in response to PPDj was high in two animals vaccinated with Montanide^™^ ISA 61 VG adjuvanted vaccination when compared to the other adjuvant groups (Fig 2F).

### Significant vaccine antigen-specific IgG1 after Montanide ISA 61 VG and CAF09 immunization

**Fig 3:**
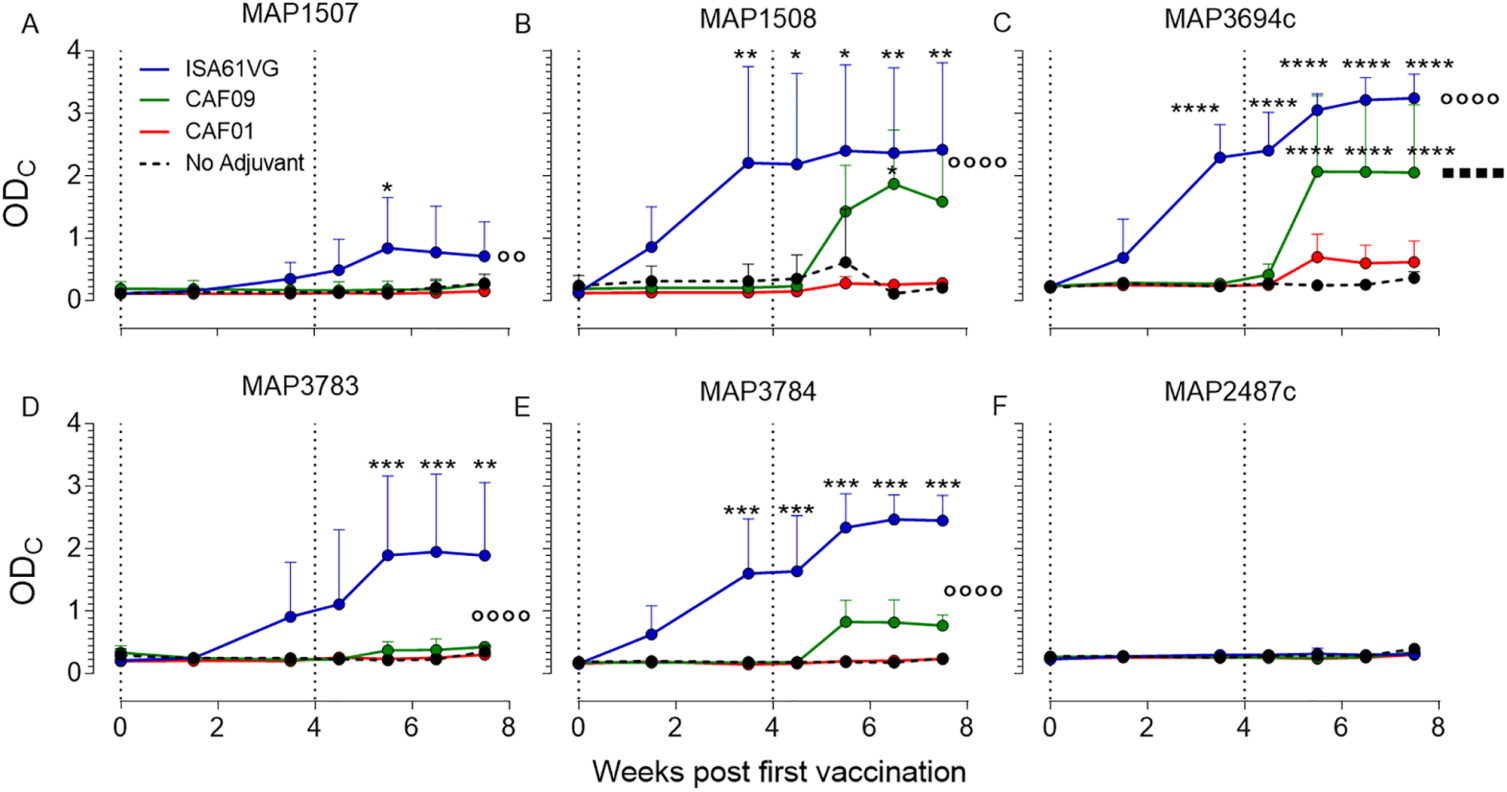
Strong humoral immune responses after CAF09 and ISA61 VG immunization. The single-antigen specific serum IgG1 level was measured in individual immunized animals. After capturing IgG1 specific for the vaccine antigens MAP1507, MAP1508, MAP3694c, MAP3783, MAP3784 or the control antigen MAP2487c the bound amount of IgG1 was measured by an in-house enzyme-linked immunosorbent assay ELISA. (**A-F**). The calibrated OD means ± SD for three calves per group are shown for all time points. Statistical analysis: two-way analysis of variance (ANOVA) and Tukey’s post-test. Black stars indicate significant difference relative to the non-adjuvant group. Circles and squares indicate differences between the curves for the adjuvanted and non-adjuvanted groups *p < 0.05, **p < 0.01, ***p < 0.001.

Using serum samples collected in parallel with the blood samples above, we measured the level of IgG1 in serum from immunized animals that was specific for the five individual vaccine antigens and included the MAP2487c protein as a negative control (Fig 3A–3F). The IgG1 serum data support and strengthen the cell-mediated pattern found in the vaccination groups by ELISPOT and whole blood IFN-γ assays. Antigen-specific IgG1 levels in Montanide ISA 61 VG group were significantly higher than in the non-adjuvant control, the CAF01-, or the CAF09-vaccinated group (p<0.01) for all vaccine antigens tested except MAP1507, which seems to be the least immunogenic of the vaccine proteins. As observed with the cell-mediated response, the IgG1 levels in the CAF09-adjuvanted group increased significantly after the second immunization. This was especially true for MAP1508 and MAP3694c where the specific IgG1 levels were significantly higher than for the non-adjuvant control group (p<0.05).

### Cationic liposomes induces polyfunctional memory CD4^+^ T cells

**Fig 4:**
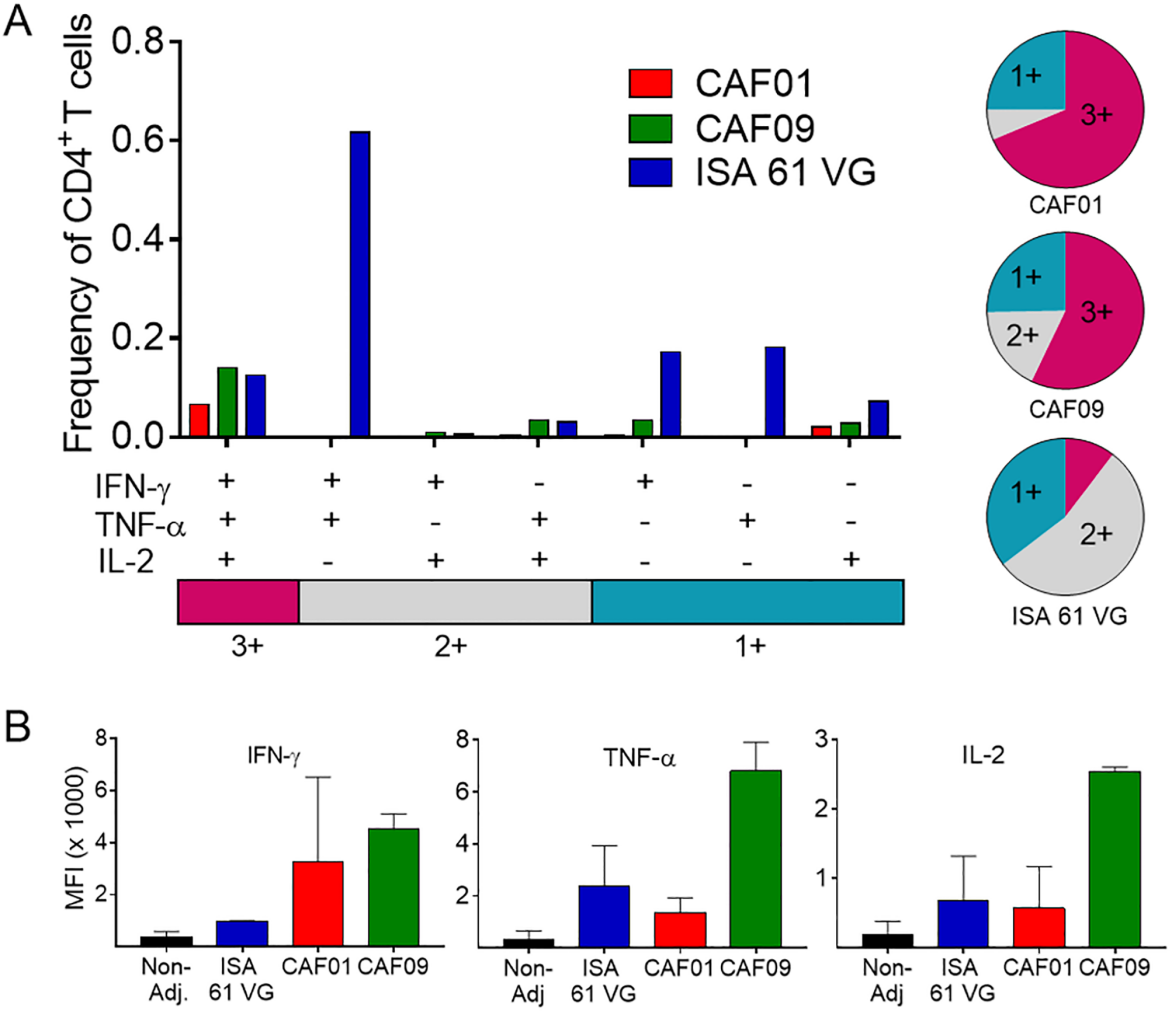
Large frequency of memory CD4^+^ T cells after CAF09 immunization. PBMCs isolated from vaccinated animals 7½ weeks after first immunization were stimulated with vaccine antigens (fusion protein + MAP3694c) and surface stained for the CD4 receptor and intracellular localized IFN-γ, TNF-α and IL-2 cytokines. Boolean gating was used to determine the frequencies of the seven possible CD4^+^ T cell subpopulations expressing any combination of the IFN-γ, TNF-α and IL-2 cytokines. The frequencies are shown for each of the three vaccination groups after subtraction of background from non-stimulated adjuvant controls. Pie charts illustrate the fraction of CD4^+^ T cell expressing three cytokines (3+), any two cytokines (2+), or any one cytokine (1+) for each vaccination group (CAF01, CAF09, and ISA 61 VG). Pie chart color-coding and the subpopulation association for each color is shown below the bar graph (**A**). The mean fluorescence intensity (MFI) for the IFN-γ-, TNF-α-, or IL-2 producing CD4+ T cells in each vaccination group was calculated as a mean of all cytokine producing cell regardless if a cell produce one, two or three of the measured cytokines. Bars represent mean ± SD of three calves. Statistical analysis: unpaired t test ***p < 0.001 (**B**).

Having shown that protein immunization with Montanide ISA 61 VG and CAF09, and to a lesser degree CAF01, can induce a sustained cell-mediated and humoral immune response we subsequently characterized the phenotype of the antigen-specific CD4^+^ T cells generated after immunization, as polyfunctional CD4+ T cells have been associated with vaccine-induced protection in mycobacterial infections [17, 18]. After staining for surface markers and intracellular cytokines we used flow cytometry and a gating strategy to measure the number and frequency of antigen-specific CD4^+^ T cells and characterized their ability to secrete one or more of the effector cytokines IFN-γ, TNF-α, and IL-2 at the single cell level (Gating in S1 Fig). With this gating strategy, it is possible to determine the functionality of antigen-specific CD4+ T cells with respect to their expression of IFN-γ, TNF-α, and IL-2 in each of the vaccination groups (Fig 4A). Immunization with antigens formulated in CAF01 or CAF09 adjuvant induced primarily polyfunctional T cell population consisting of triple cytokine producing memory CD4^+^ T cells (IFN-γ^+^ TNF-α^+^ IL-2^+^) and a lower frequency of double (IFN-γ^+^ TNF-α^+^) and single (IFN-γ^+^ or TNF-α^+^) cytokine positive effector CD4+ T cells. In agreement with the stronger responses measured above, there was a higher frequency of vaccine-specific CD4+ T cells in the CAF09-immunized compared to CAF01-immunized animals, but also a relatively larger portion of more differentiated single and double cytokine positive effector cells. Following Montanide ISA 61 VG immunization, we found the opposite. The animals had a high frequency of terminally differentiated effector CD4+ T cells, and especially cells producing two cytokines (IFN-γ^+^ TNF-α^+^), but there was also a considerable frequency of IFN-γ single producers and triple positive memory T cells. To compare the average amount of each cytokine that was produced by an individual cell, regardless if the cell was a single- or multi-cytokine producer, we measured the median fluorescence intensities (MFI) for each of the cytokines. For all three cytokines, CD4^+^ T cells from CAF09-immunized animals produced the most cytokine per cell (Fig 4B). This was also reflected in the results when we compared the level of IFN-γ-, TNF-α-, or IL-2 per cell in each of the CD4+ T cell cytokine producing subgroups (data in S2 Fig). In the triple positive polyfunctional T cells, cytokine production per cell was highest in T cells from CAF09 immunized animals for all three cytokines measured and particularly for TNF-α in the TNF-α IL-2 double producing T cell population. Thus, the flow cytometry data confirms the IFN-γ ELISPOT and ELISA data showing that Montanide ISA61 VG predominantly induces a strong response because it activates a high number of CD4^+^ T cells. In contrast, CAF09 activates fewer CD4^+^ T cells, but these produce more cytokines and a higher amount of each individual cytokine per cell.

### Subunit immunization does not interfere with tuberculosis or paratuberculosis diagnosis

The majority of the commercially available vaccines against mycobacterial infections interfere with skin test and ELISA-based diagnoses. To address this issue we tested all animals used in the study with standard tuberculin skin test as well as with a commercial indirect ELISA kit measuring anti-MAP antibodies. Using the standard guidelines for interpreting the reactors in tuberculin skin test, we found that all animals from the vaccinated groups were negative (<2 mm increase in skin thickness 72 hours post injection; data not shown). Furthermore, all animals used in the study were seronegative in a commercial ID Screen^®^ paratuberculosis assay using indirect ELISA to measure IgG responses in serum. For serum samples collected 7½ weeks after the first vaccination we found an S/P ratio range between 1.36 and 17.76 with a mean value of 2.33 for all animals, far below the cut-off value of ≥ 70 percent.

## Discussion

Lymphocytes, in particular CD4 T cells, are believed to be critically required for host defense against intracellular mycobacterial pathogens in both man and animals [19–21]. The liposome-based CAF01 adjuvant can induce strong CD4 T cell responses in several species including humans and pigs, but in cattle the systemic response has been quite low and a significant increase in dose will make a future vaccine prohibitively expensive [10, 11]. Alternatively, quality and strength of the vaccine-mediated immune response can be directed by the nature and dose of immune-stimulating component(s) in the vaccine formulation [22–24]. Thus, with the aim of improving the immunogenicity of liposome-based adjuvants, we evaluated immunostimulators using bovine monocytes and macrophages *in vitro* to reinforce the potential use of next generation liposome-based adjuvants in cattle.

Macrophages and dendritic cells are located throughout the body to capture and internalize invading pathogens. As part of the innate immune system, they are first line of defense against infections and activators of the adaptive immune system through antigen presentation and co-stimulation of lymphocytes. Circulating naïve monocytes and macrophages present pattern recognition receptors (PRRs), which recognize molecules (pathogen-associated molecular patterns, PAMPs) that are broadly shared by pathogens but distinguishable from host molecules. PRRs include Toll-like receptors (TLR), RIG like receptors (RLR), C-type lectin receptors (CLR), and NOD-like receptors (NLR). PRR engagement leads to monocyte and macrophage activation, increased antigen presentation, and release of cytokines and chemokines that orchestrate the ensuing adaptive immune response. A trend in modern vaccine development is to mimic the pathogen danger signal by incorporating one or more PRR ligands into the adjuvant formulation, thereby providing the immunogenicity of a live-attenuated vaccine without the associated safety issues. In vitro studies have shown that PRR engagement affects the APC’s secretion of cytokines, including IL-12, IL-6, and TNF-α. In this study, we have used secretion of cytokine IL-6 after incubation with ligands as a proxy for monocyte/macrophage activation. TDB (trehalose 6′6-dibehenate) is a synthetic analogue of mycobacterial cord factor or trehalose dimycolate (TDM) with less toxicity but retained adjuvanticity *in vivo* [25]. TDB is a PRR-ligand that activates cells through the TLR-independent Syk-Card9 signaling pathway by binding to and upregulating the Mincle receptor on the cell surface [26, 27]. The TDB activation of bovine monocytes and macrophages followed an inverted U-shaped dose-response curve with a narrow range of lipid densities activating the cells. The highest coating density did not give the strongest signal most likely because, even though less toxic than TDM, TDB is toxic to APCs at high lipid densities. Monomycolyl glycerol (MMG) is another mycobacterial cell-wall lipid that signals via the Mincle receptor. MMG has been reported to induce a Th1 immune response [28] and, based on enhanced expression of activation markers and the release of proinflammatory cytokines, to be a better stimulator of human DCs than TDB [15]. When bovine monocytes and macrophages were stimulated with an MMG lipid layer the cells were specifically activated but cytokine release was lower than for TDB and only one coating density resulted in measurable cell activation. Due to a very low solubility in aqueous solutions, the polystyrene surface was coated with TDB and MMG lipids only allowing a 2-D surface for activation [29]. We might have obtained higher cytokine IL-6 release by sonicating the lipids in the medium. Nevertheless, the in vitro stimulation data shows that both TDB and MMG has immunostimulatory activity on bovine monocytes and macrophages and the Mincle receptor and Syk—Card9 pathway is a rational target for vaccine development in cattle. Poly(I:C) is a synthetic analog that is structurally similar to double-stranded RNA and a stimulant of the endosomal located TLR3 and cytosolic MDA-5 and RIG-1 [30, 31]. Poly(I:C) targets a different signaling pathway than TDB and MMG, but Poly(I:C) and its derivatives have consistently been shown to be among the strongest Th1-inducing immunomodulators [32, 33]. TLR3 signaling is MyD88-independent and utilizes adaptor protein toll/interleukin-1 receptor (TIR) domain-containing adaptor inducing IFN-β (TRIF) to induce the release of inflammatory cytokinesis and a type 1 IFN response [34, 35]. We tested Poly(I:C) in two *in vitro* concentrations, and only the highest concentration (50 μg/ml) activated the monocytes and monocyte-derived macrophages. While this is a high Poly(I:C) concentration the specific cell activation showed that also the TLR3/TRIF pathway can be exploited in bovine vaccine development. During natural infections, microbial derived PRR agonists do not function individually; rather they synergize to manipulate the immune response via activation of APCs. *In vivo* and *in vitro* studies have found that triggering of several PRRs simultaneously can induce diverse innate immune responses. Some combinations enhanced cytokine and chemokine production by APCs others impaired APCs activation [23, 36, 37]. Specifically it has been shown that combining the TLR3 agonist Poly(I:C) with the TLR6/TLR2 agonist MALP-2 increased the number of activated T cells [36]. Adding a third agonist, the TLR9 agonist CpG, and a HIV peptide, induced higher-functional avidity T cells than with single or double ligand combinations and an effective response against viral challenge [24]. Using a mycobacterial vaccine, it was shown that combining a TLR4 and TLR9 agonist enhanced the vaccine-specific T cell response and increased protection against challenge [37]. These synergistic effects of multiple receptor ligations have primarily been reported when TLR agonists activating the TRIF pathway have been combined with TLR agonists activating the MyD88 pathway [38]. Our results extend these observations by demonstrating a synergistic effect on *in vitro* activation of bovine monocytes and macrophages after simultaneously targeting two different types of PRRs and their signaling pathways. Our *in vitro* data do not select between TDB+Poly(I:C) and MMG+Poly(I:C). Previous studies have shown that DDA-Poly(I:C) combined with TDB or MMG-1 can be formulated to stable adjuvants (CAF05 and CAF09, respectively) which both can induce strong CD4+ T cell responses of the Th1/Th17 phenotype [39–41]. However, MMG-1 is structurally simpler and easier to manufacture than TDB, the CAF09 liposomes are smaller, less polydispersed and more stable than CAF05 liposomes, and CAF09 was therefore selected for further study [40]. *In vitro*, bovine monocytes and macrophages were activated by CAF09 doses containing significantly less MMG and Poly(I:C) than needed for activation without the DDA liposomes. We suggest this is due to a dual function of the DDA liposomes. They keep the MMG glycolipid in dispersion for better presentation to the Mincle receptor on the cell surface and increase the cellular uptake of the liposomes for optimal Poly(I:C) activation via the endosomal located TLR3. Because of the significant increase in activation efficacy, we decided to test CAF09 in cattle.

Several Montanide adjuvants have been shown to induce strong and long-lasting humoral and cell mediated immune responses in cattle, and Montanide ISA 61 VG was therefore included as a positive control for vaccine specific IgGs and cell-mediated immune responses after immunization [10, 42]. Montanide ISA 61 VG consists of squalene emulsified with mannide mono-oleate and belongs to the group of incomplete Freund’s adjuvants (IFA). Despite well advanced into Phase I and II vaccine trials, the only information regarding the mechanism of action for Montanide based vaccines is a depot effect at the site of injection, enabling slow antigen release. However, new insights have been made into the mode of action of IFA which could also explain the action mechanisms of Montanide, including more effective transport of the antigens to the lymphatic system and providing a complex set of signals to the innate immune system, thereby directing and orchestrating development and function of antigen-specific T and B lymphocytes [43]. As discussed above, CAF01’s activation mechanism via TDB is well described and, like Montanide, it creates a long-lasting depot at the site of injection and increases the monocyte and macrophage influx to the site of injection [44]. In cattle, CAF01-adjuvanted vaccines induce low immune responses, which is surprising since the Mincle receptor is phylogenetic conserved and expressed on bovine antigen-presenting cells. However, in transfected macrophages expressing either the human or the murine Mincle receptor a few amino acid differences between the Mincle sequences determined if the cells were effectively activated by the Mincle ligand MMG (Monomycoloyl glycerol) or not [45]. Thus, it is possible that sequence polymorphism is the main culprit for the lack of strong cell—mediated immune responses in CAF01 vaccinated cattle and potentially other ruminants. In this study, CAF01 served as a benchmark for the CAF09 based vaccine and in line with previous studies it promoted low humoral and cell mediated vaccine specific responses. Montanide ISA 61 VG induced both a strong cell-mediated and humoral vaccine-specific immune responses after one immunization but the second immunization did not boost the number of IFN-γ producing cells or the level of released IFN-γ cytokine. CAF09-induced humoral responses were stronger than for CAF01 but weaker than for Montanide ISA 61 VG and detectable only after the second immunization. After one immunization, CAF09-induced approximately the same number of vaccine-specific IFN-γ secreting cells as Montanide ISA 61 VG but, based on secreted IFN-γ levels, the cells produced less IFN-γ per cell, and it took a second immunization before they reached the same cytokine secretion level as in the Montanide ISA 61 VG group. We know that polyfunctional T cells produce a higher amount of cytokine per cell than single functional T cells and it was thus possible that repeated CAF09 immunization improved the quality of the immune response [46]. To determine the quality and multifunctionality of vaccine-specific CD4^+^ T cells, we measured IFN-γ, TNF-α and IL-2 cytokine production in individual cells. IFN-γ is a key effector molecule that synergizes with TNF-α in activating infected APCs, allowing the cells to better control intracellular infection [47, 48]. IL-2 is needed for T cell expansion, differentiation, and long-term cell survival and is used as a marker for less differentiated central memory T cells [46, 49, 50]. In mice, the CD4+ T cell population induced by CAF01- or CAF09-adjuvanted vaccines have been shown to be dominated primarily by TNF-α^+^ IL-2^+^ and secondarily by IFN-γ^+^ TNF-α^+^ IL-2^+^ multifunctional CD4+ T cells. These T cells have a large proliferative potential, they are efficiently maintained for more than one year, and provide long-term protective immunity [18, 51]. Although TDB and MMG derives from *Mycobacterium tuberculosis*, the CAF01 and CAF09 induced CD4+ memory T cell population differ significantly from the effector T cell population (IFN-γ^+^ and TNF-α^+^ single or double cytokine producers) dominant in *Mycobacterium tuberculosis*-infected animals [6]. In cattle, the cytokine expression profile in CD4+ T cells from Montanide ISA 61 VG-immunized animals was very similar to the effector populations seen after a *Mycobacterium tuberculosis* infection in mice, with a large population of IFN-γ^+^ TNF-α^+^ effector cells. In the CAF01 and CAF09 immunized groups, the vaccine-specific CD4+ T cells were primarily triple positive T cells (IL-2^+^ IFN-γ^+^ TNF-α^+^) but, although less dominant, there was an equally fraction of these polyfunctional T cells in the Montanide ISA 61 VG-immunized animals. Cytokine production per cell in this important subgroup of CD4+ T cells was highest in CAF09 immunized animals for each of the three cytokines. In mice the dominant vaccine specific T cell subpopulation is the less differentiated IL-2^+^ TNF-α^+^ cells but this subgroup was less prominent in our study most likely because murine cells typically are isolated from lymph nodes, spleens or the site of infection whereas the bovine data are from peripheral blood cells [18, 51].

One important consideration for mycobacterial vaccines intended for use in cattle is that the vaccine does not interfere with standard tuberculin-based skin testing but allows concurrent vaccination and stamping-out procedures, *i*.*e*. it has to be possible to discriminate infected from vaccinated animals (DIVA). Here and in previous work with recombinant MAP antigens, we did not find any animals in any of the vaccination groups that were positive to the tuberculin skin tests or an ELISA-based paratuberculosis test detecting IgG antibodies [11]. We did however find higher PPDj-specific IFN-γ responses in blood from animals vaccinated with Montanide ISA 61 VG adjuvanted antigens relative to the other adjuvant groups and that could potentially interfere with PPDj based blood CMI assays. Currently, we do not have a correlate of protection against mycobacterial infection in any species, but it appears that CAF09 is an attractive alternative to oil-in-water based adjuvants in cattle, not only for mycobacterial infections but also for a broader use.

The demand for a more precise tailoring of the immune response will drive adjuvant development based on combining PAMPs. In this regard, the two-step *in vitro*/*in vivo* approach is relevant for preselecting and testing agonist combinations and adjuvants. Combined with a dissection of the signaling pathways and their effects on host immune cells in different species, it will advance our understanding of the molecular mechanisms involved in the induction of adaptive immune responses.

The next generation CAF09 based adjuvant, could *e*.*g*. include a TLR agonist that signals through MyD88 or PRRs such as DC-SIGN, BDCA2, DCIR and MICL that induces signaling pathways modulating TLR-induced gene expression at the transcriptional or post-transcriptional level. These receptors do not induce immune responses on their own, but modulate signaling pathways induced by other PRRs and could provide the flexibility and variability in cytokine expression that is needed to combat different pathogens [52–54]. CD14 is a cofactor for several TLRs and activation of CD14 could synergistically stimulate multiple TLR for a more potent adaptive immunity. However, since, CD14 induces an endocytosis pathway that delivers TLR4 to endosomes and evasion of CD14-dependent endocytosis is a survival strategy for intracellular bacterial pathogens CD14 activation could be especially relevant to include in a therapeutic vaccine [55].

In summary, combining a distinct TLR and CLR agonist into an adjuvanted subunit vaccine increased the magnitude of both the cellular Th1 response and the humoral antibody response directly in a vaccine target species without compromising the polyfunctionality of the vaccine-specific CD4+ T cells in the blood.

New vaccines that harness these powerful signaling properties of PRRs will allow us to tailor immune responses against a specific pathogen or disease.

## Supporting information

S1 Fig Gating strategy for cytokine producing CD4 cells. (TIF)

S2 Fig Level of cytokine production in subgroups of cytokine producing CD4 T cells. The mean fluorescent intensity (MFI) was measured in CD4 T cell subgroups—grouped based on their cytokine expression profiles—for each of the three cytokines in PBMCs isolated from ISA 61VG (blue), CAF01 (red) or CAF09 (green) vaccinated animals. Blood was drawn 7½ weeks after first immunization.
(TIF)

S1 Table IL-6 production after in vitro stimulation. The concentration of cytokine IL-6 in the supernatant is given in pg/ml.
(XLSX)

S2 Table Immune responses in blood from vaccinated and control animals. IFN-γ production or the number of IFN-γ producing cells was measured by ELISA or ELISpot in blood from vaccinated and non-vaccinated control animals. Numbers of IFN-γ positive cells are given per 10^6^ PBMCs and cytokine production as pg/ml.
(XLSX)

S3 Table IgG1 levels against single antigens in blood from vaccinated and control animals. Optical densities (493 nm) are given after a reference subtraction (649-nm).
(XLSX)

S4 Table Flow cytometry data. Frequencies of specific CD4 T cells divided into subgroups based on cytokine expression profiles and MFIs for the three cytokines measured.
(XLSX)
