# Supplementary figures and images for: Targeting the Mincle and TLR3 receptor using the dual agonist cationic adjuvant formulation 9 (CAF09) induces humoral and polyfunctional memory T cell responses in calves
Source: PLoS One. 2018 Jul 31;13(7):e0201253. doi: 10.1371/journal.pone.0201253 (PMC6067743; doi:10.1371/journal.pone.0201253)

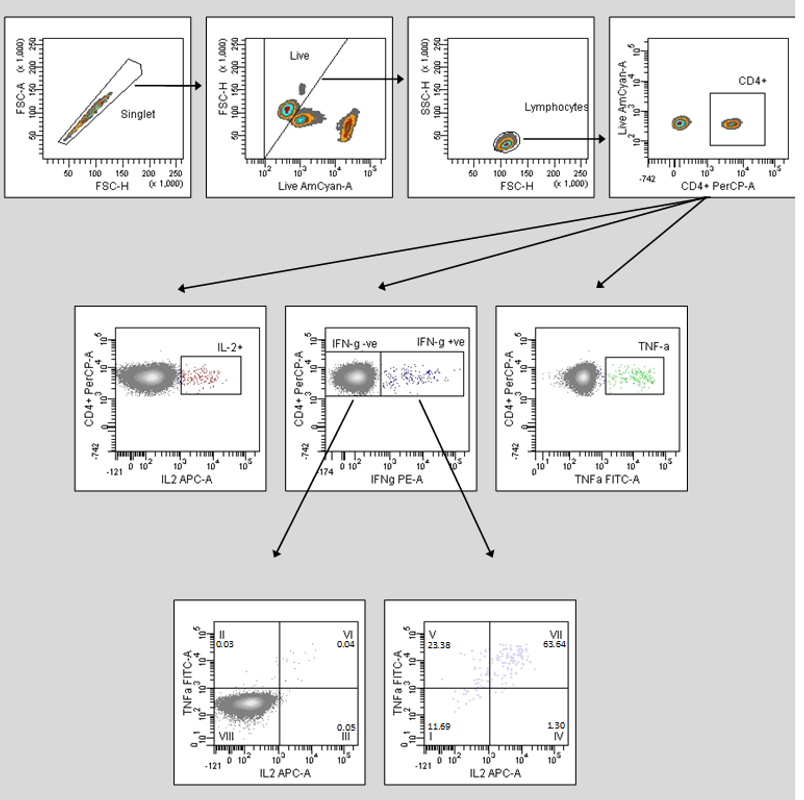

Supplement: S1 Fig — (TIF) [file pone.0201253.s001.tif]

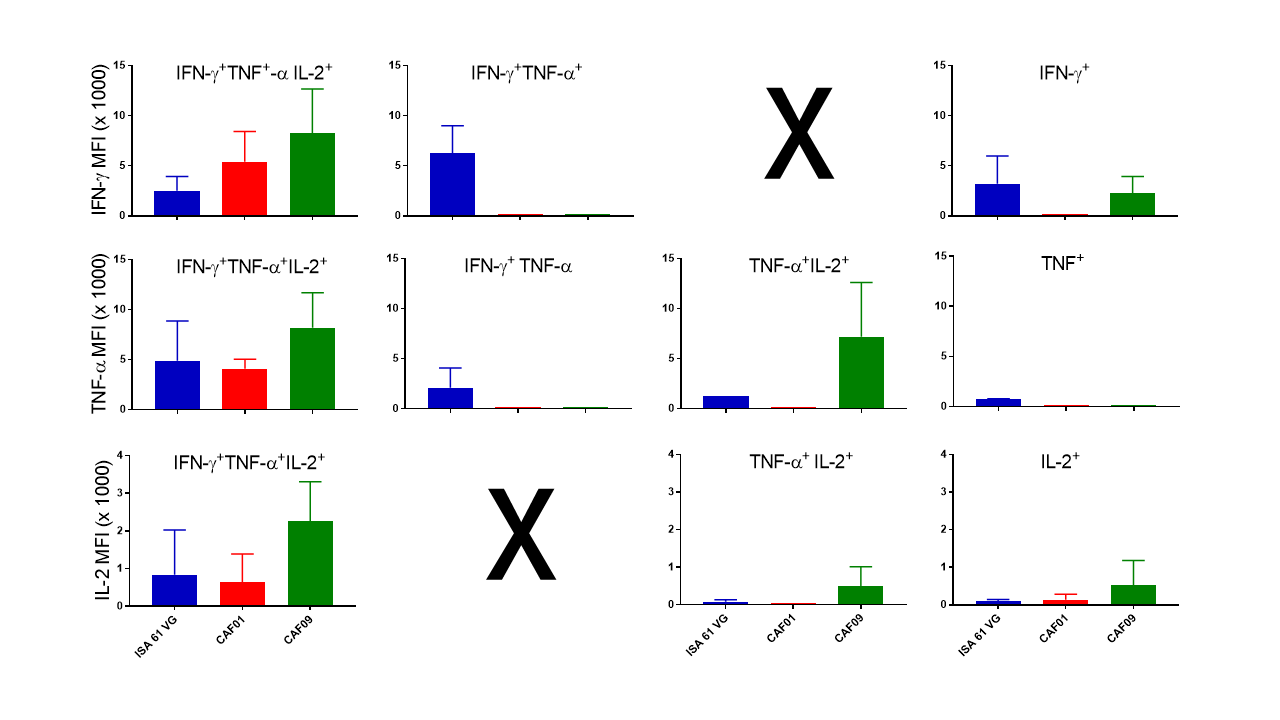

Supplement: S2 Fig — The mean fluorescent intensity (MFI) was measured in CD4 T cell subgroups—grouped based on their cytokine expression profiles—for each of the three cytokines in PBMCs isolated from ISA 61VG (blue), CAF01 (red) or CAF09 (green) vaccinated animals. Blood was drawn 7½ weeks after first immunization. (TIF) [file pone.0201253.s002.tif]
